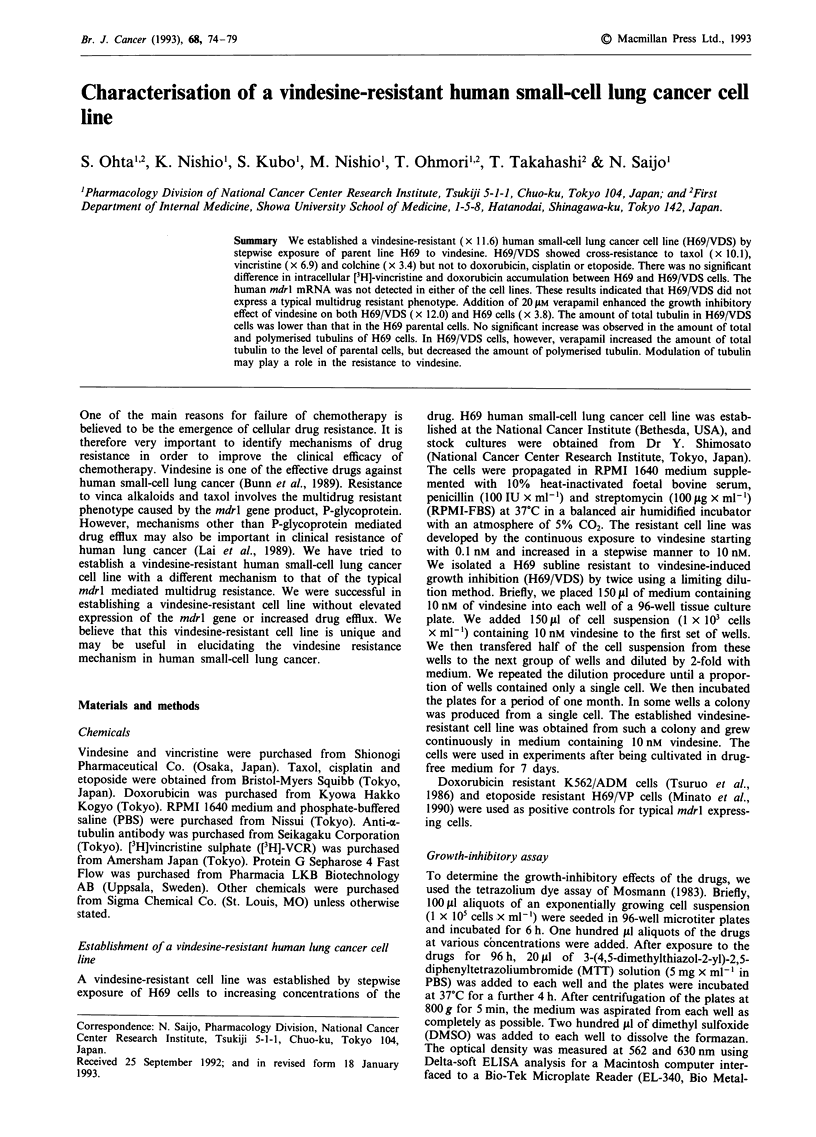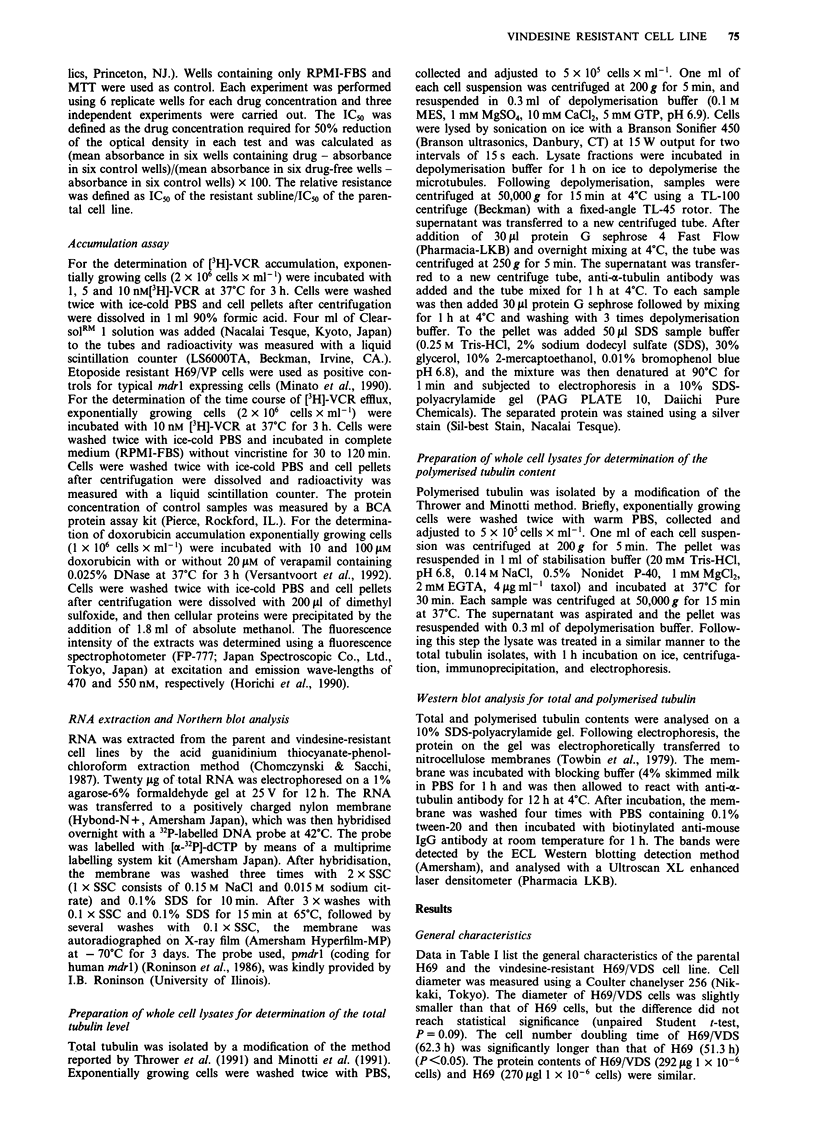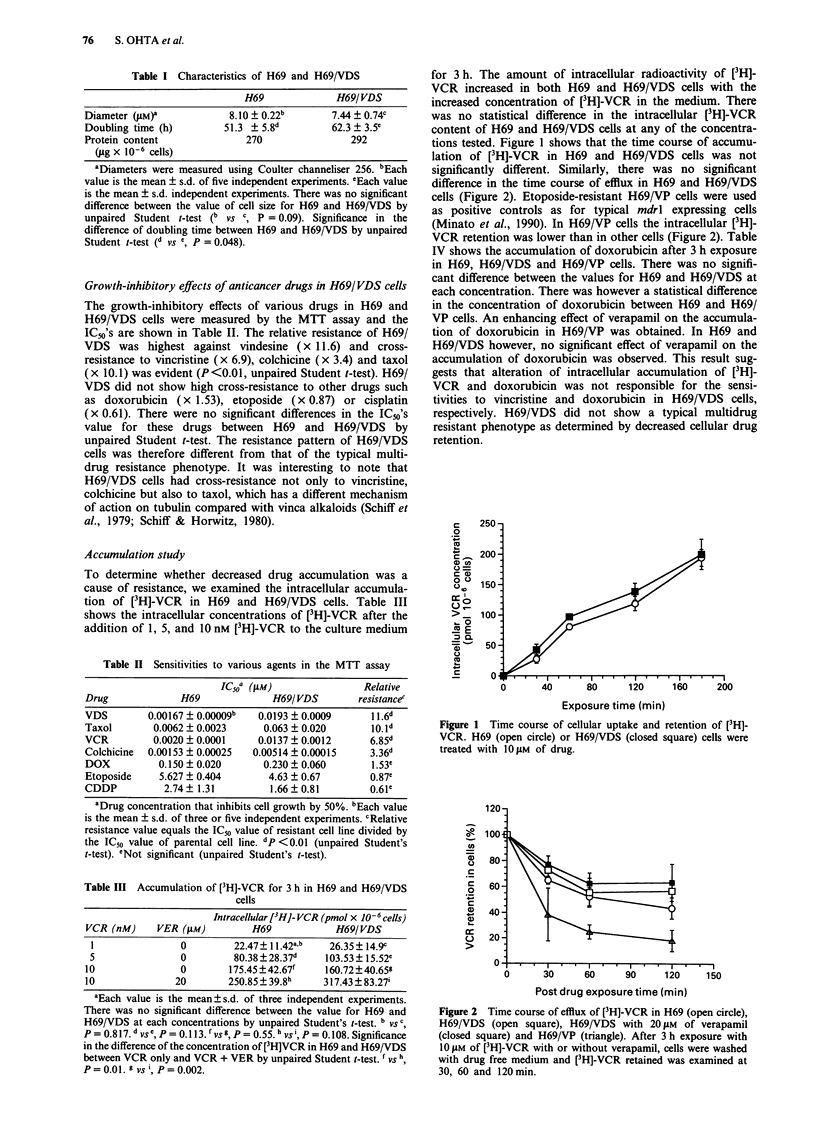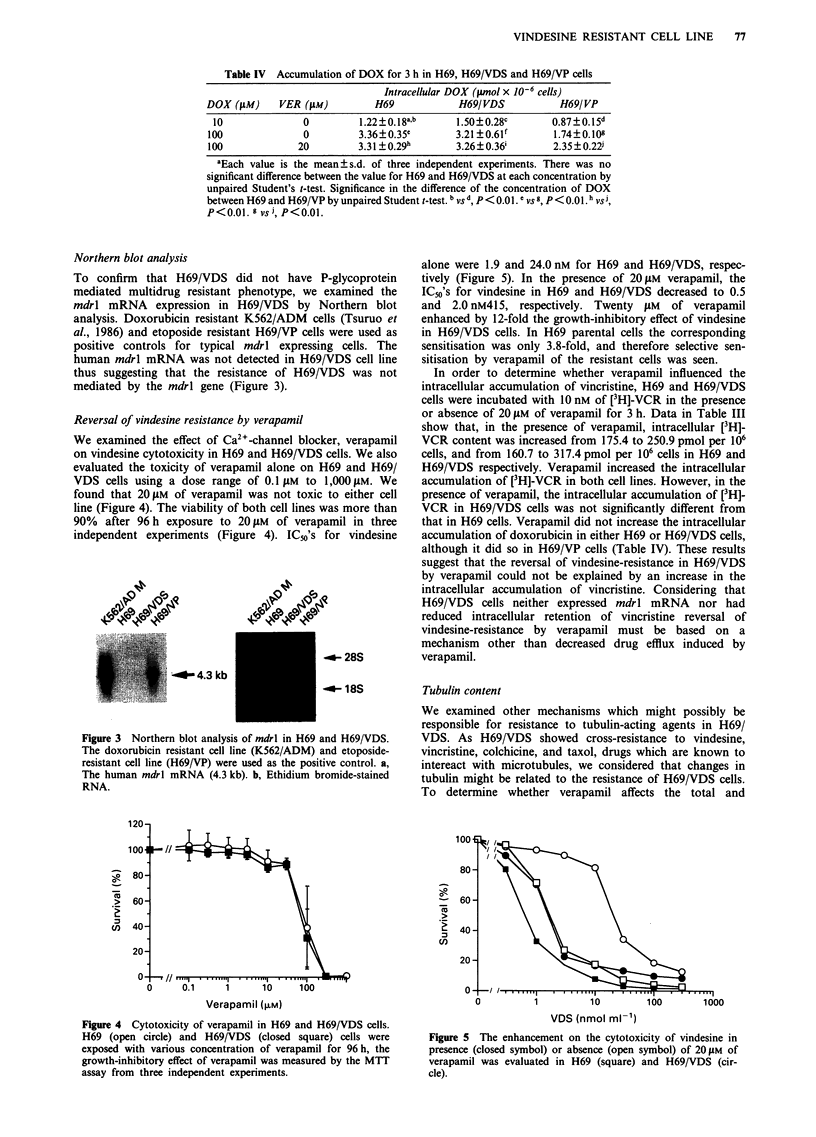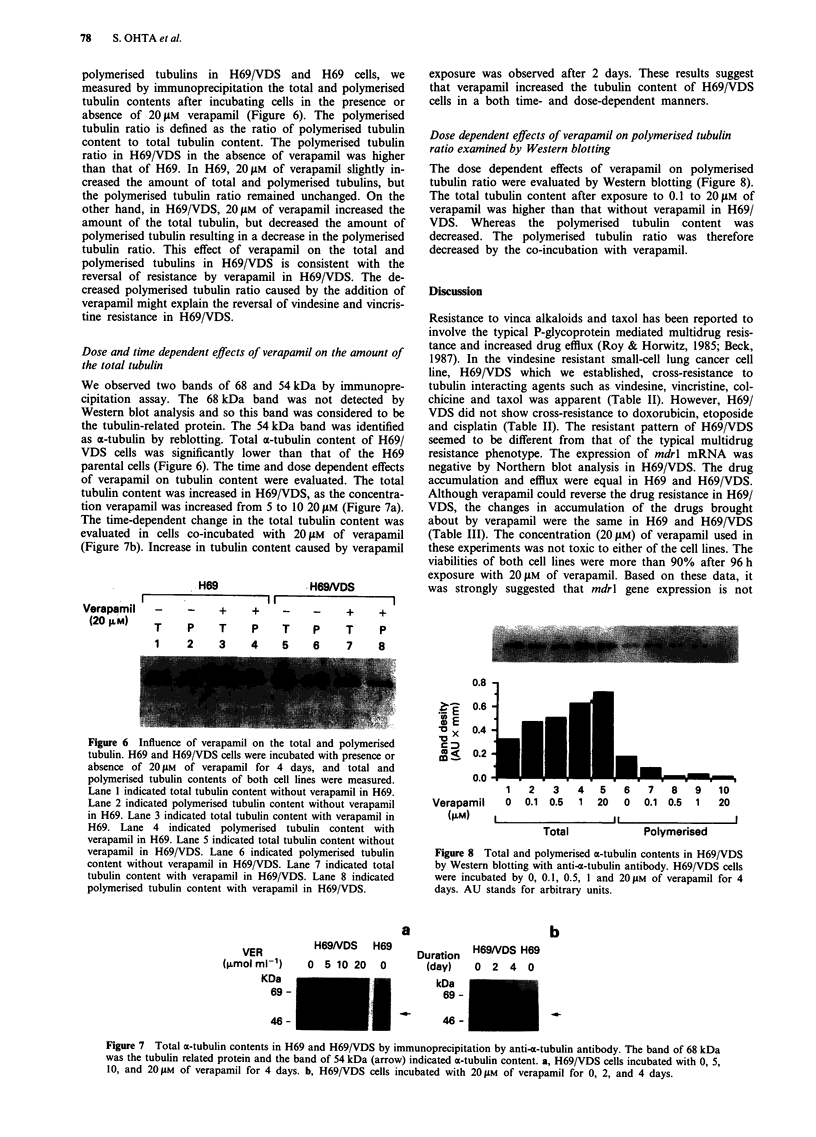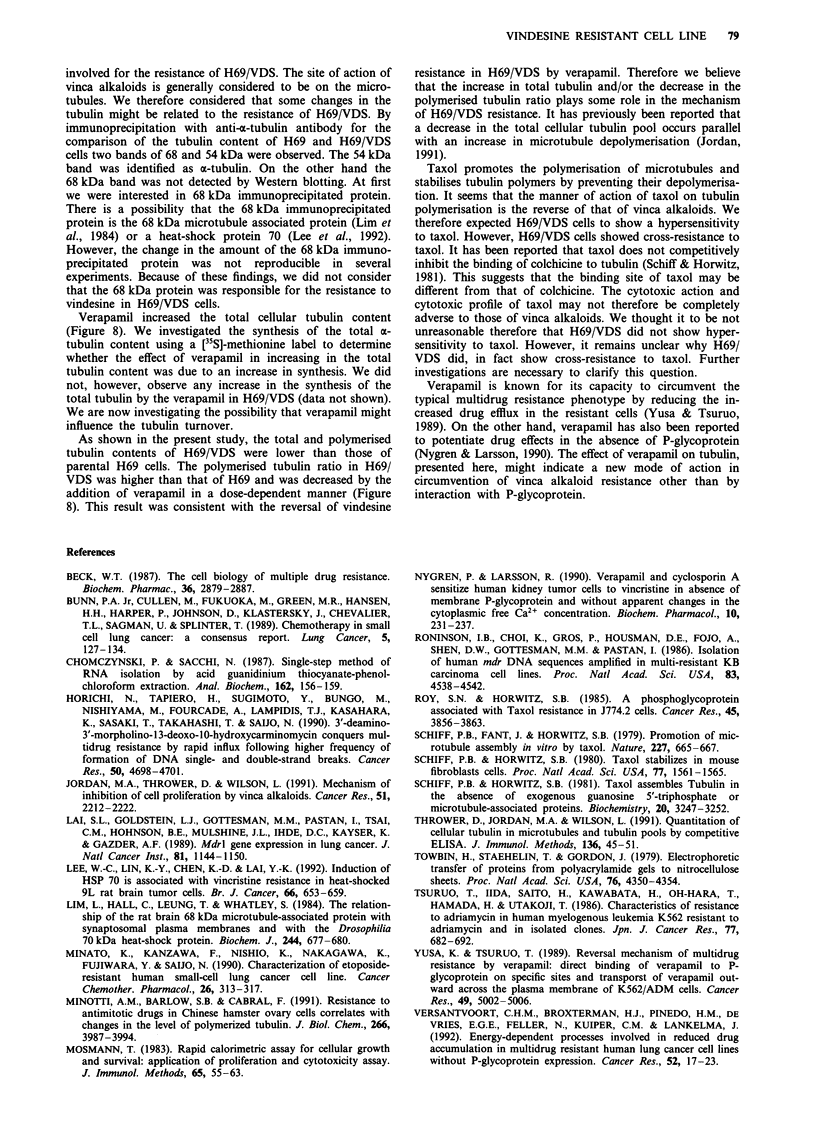# Characterisation of a vindesine-resistant human small-cell lung cancer cell line.

**DOI:** 10.1038/bjc.1993.289

**Published:** 1993-07

**Authors:** S. Ohta, K. Nishio, S. Kubo, M. Nishio, T. Ohmori, T. Takahashi, N. Saijo

**Affiliations:** Pharmacology Division of National Cancer Center Research Institute, Tokyo, Japan.

## Abstract

**Images:**


Br. J. Cancer (1993), 68, 74-79 © Macmillan Press Ltd., 1993

### Characterisation of a vindesine-resistant human small-cell lung cancer cell line

S. Ohta[1,2], K. Nishio[1], S. Kubo[1], M. Nishio[1], T. Ohmori[1,2], T. Takahashi[2] & N. Saijo[1]

[1]*Pharmacology Division of National Cancer Center Research Institute, Tsukiji 5-1-1, Chuo-ku, Tokyo 104, Japan; and* [2]*First Department of Internal Medicine, Showa University School of Medicine, 1-5-8, Hatanodai, Shinagawa-ku, Tokyo 142, Japan.*

**Summary** We established a vindesine-resistant (× 11.6) human small-cell lung cancer cell line (H69/VDS) by stepwise exposure of parent line H69 to vindesine. H69/VDS showed cross-resistance to taxol (× 10.1), vincristine (× 6.9) and colchine (× 3.4) but not to doxorubicin, cisplatin or etoposide. There was no significant difference in intracellular [$^3$H]-vincristine and doxorubicin accumulation between H69 and H69/VDS cells. The human *mdr*1 mRNA was not detected in either of the cell lines. These results indicated that H69/VDS did not express a typical multidrug resistant phenotype. Addition of 20 µM verapamil enhanced the growth inhibitory effect of vindesine on both H69/VDS (× 12.0) and H69 cells (× 3.8). The amount of total tubulin in H69/VDS cells was lower than that in the H69 parental cells. No significant increase was observed in the amount of total and polymerised tubulins of H69 cells. In H69/VDS cells, however, verapamil increased the amount of total tubulin to the level of parental cells, but decreased the amount of polymerised tubulin. Modulation of tubulin may play a role in the resistance to vindesine.

One of the main reasons for failure of chemotherapy is believed to be the emergence of cellular drug resistance. It is therefore very important to identify mechanisms of drug resistance in order to improve the clinical efficacy of chemotherapy. Vindesine is one of the effective drugs against human small-cell lung cancer (Bunn *et al.*, 1989). Resistance to vinca alkaloids and taxol involves the multidrug resistant phenotype caused by the *mdr*1 gene product, P-glycoprotein. However, mechanisms other than P-glycoprotein mediated drug efflux may also be important in clinical resistance of human lung cancer (Lai *et al.*, 1989). We have tried to establish a vindesine-resistant human small-cell lung cancer cell line with a different mechanism to that of the typical *mdr*1 mediated multidrug resistance. We were successful in establishing a vindesine-resistant cell line without elevated expression of the *mdr*1 gene or increased drug efflux. We believe that this vindesine-resistant cell line is unique and may be useful in elucidating the vindesine resistance mechanism in human small-cell lung cancer.

### Materials and methods

### *Chemicals*

Vindesine and vincristine were purchased from Shionogi Pharmaceutical Co. (Osaka, Japan). Taxol, cisplatin and etoposide were obtained from Bristol-Myers Squibb (Tokyo, Japan). Doxorubicin was purchased from Kyowa Hakko Kogyo (Tokyo). RPMI 1640 medium and phosphate-buffered saline (PBS) were purchased from Nissui (Tokyo). Anti-α-tubulin antibody was purchased from Seikagaku Corporation (Tokyo). [$^3$H]vincristine sulphate ([$^3$H]-VCR) was purchased from Amersham Japan (Tokyo). Protein G Sepharose 4 Fast Flow was purchased from Pharmacia LKB Biotechnology AB (Uppsala, Sweden). Other chemicals were purchased from Sigma Chemical Co. (St. Louis, MO) unless otherwise stated.

### *Establishment of a vindesine-resistant human lung cancer cell line*

A vindesine-resistant cell line was established by stepwise exposure of H69 cells to increasing concentrations of the drug. H69 human small-cell lung cancer cell line was established at the National Cancer Institute (Bethesda, USA), and stock cultures were obtained from Dr Y. Shimosato (National Cancer Center Research Institute, Tokyo, Japan). The cells were propagated in RPMI 1640 medium supplemented with 10% heat-inactivated foetal bovine serum, penicillin (100 IU × ml$^{-1}$) and streptomycin (100 µg × ml$^{-1}$) (RPMI-FBS) at 37°C in a balanced air humidified incubator with an atmosphere of 5% $CO_2$. The resistant cell line was developed by the continuous exposure to vindesine starting with 0.1 nM and increased in a stepwise manner to 10 nM. We isolated a H69 subline resistant to vindesine-induced growth inhibition (H69/VDS) by twice using a limiting dilution method. Briefly, we placed 150 µl of medium containing 10 nM of vindesine into each well of a 96-well tissue culture plate. We added 150 µl of cell suspension (1 × 10$^3$ cells × ml$^{-1}$) containing 10 nM vindesine to the first set of wells. We then transfered half of the cell suspension from these wells to the next group of wells and diluted by 2-fold with medium. We repeated the dilution procedure until a proportion of wells contained only a single cell. We then incubated the plates for a period of one month. In some wells a colony was produced from a single cell. The established vindesine-resistant cell line was obtained from such a colony and grew continuously in medium containing 10 nM vindesine. The cells were used in experiments after being cultivated in drug-free medium for 7 days.

Doxorubicin resistant K562/ADM cells (Tsuruo *et al.*, 1986) and etoposide resistant H69/VP cells (Minato *et al.*, 1990) were used as positive controls for typical *mdr*1 expressing cells.

### *Growth-inhibitory assay*

To determine the growth-inhibitory effects of the drugs, we used the tetrazolium dye assay of Mosmann (1983). Briefly, 100 µl aliquots of an exponentially growing cell suspension (1 × 10$^5$ cells × ml$^{-1}$) were seeded in 96-well microtiter plates and incubated for 6 h. One hundred µl aliquots of the drugs at various concentrations were added. After exposure to the drugs for 96 h, 20 µl of 3-(4,5-dimethylthiazol-2-yl)-2,5-diphenyltetrazoliumbromide (MTT) solution (5 mg × ml$^{-1}$ in PBS) was added to each well and the plates were incubated at 37°C for a further 4 h. After centrifugation of the plates at 800 *g* for 5 min, the medium was aspirated from each well as completely as possible. Two hundred µl of dimethyl sulfoxide (DMSO) was added to each well to dissolve the formazan. The optical density was measured at 562 and 630 nm using Delta-soft ELISA analysis for a Macintosh computer interfaced to a Bio-Tek Microplate Reader (EL-340, Bio Metal-

Correspondence: N. Saijo, Pharmacology Division, National Cancer Center Research Institute, Tsukiji 5-1-1, Chuo-ku, Tokyo 104, Japan.
Received 25 September 1992; and in revised form 18 January 1993.

lics, Princeton, NJ.). Wells containing only RPMI-FBS and MTT were used as control. Each experiment was performed using 6 replicate wells for each drug concentration and three independent experiments were carried out. The $IC_{50}$ was defined as the drug concentration required for 50% reduction of the optical density in each test and was calculated as (mean absorbance in six wells containing drug − absorbance in six control wells)/(mean absorbance in six drug-free wells − absorbance in six control wells) × 100. The relative resistance was defined as $IC_{50}$ of the resistant subline/$IC_{50}$ of the parental cell line.

*Accumulation assay*

For the determination of [$^3$H]-VCR accumulation, exponentially growing cells ($2 \times 10^6$ cells × $ml^{-1}$) were incubated with 1, 5 and 10 nM[$^3$H]-VCR at 37°C for 3 h. Cells were washed twice with ice-cold PBS and cell pellets after centrifugation were dissolved in 1 ml 90% formic acid. Four ml of Clear-sol$^{RM}$ 1 solution was added (Nacalai Tesque, Kyoto, Japan) to the tubes and radioactivity was measured with a liquid scintillation counter (LS6000TA, Beckman, Irvine, CA.). Etoposide resistant H69/VP cells were used as positive controls for typical *mdr*l expressing cells (Minato *et al.*, 1990). For the determination of the time course of [$^3$H]-VCR efflux, exponentially growing cells ($2 \times 10^6$ cells × $ml^{-1}$) were incubated with 10 nM [$^3$H]-VCR at 37°C for 3 h. Cells were washed twice with ice-cold PBS and incubated in complete medium (RPMI-FBS) without vincristine for 30 to 120 min. Cells were washed twice with ice-cold PBS and cell pellets after centrifugation were dissolved and radioactivity was measured with a liquid scintillation counter. The protein concentration of control samples was measured by a BCA protein assay kit (Pierce, Rockford, IL.). For the determination of doxorubicin accumulation exponentially growing cells ($1 \times 10^6$ cells × $ml^{-1}$) were incubated with 10 and 100 μM doxorubicin with or without 20 μM of verapamil containing 0.025% DNase at 37°C for 3 h (Versantvoort *et al.*, 1992). Cells were washed twice with ice-cold PBS and cell pellets after centrifugation were dissolved with 200 μl of dimethyl sulfoxide, and then cellular proteins were precipitated by the addition of 1.8 ml of absolute methanol. The fluorescence intensity of the extracts was determined using a fluorescence spectrophotometer (FP-777; Japan Spectroscopic Co., Ltd., Tokyo, Japan) at excitation and emission wave-lengths of 470 and 550 nM, respectively (Horichi *et al.*, 1990).

*RNA extraction and Northern blot analysis*

RNA was extracted from the parent and vindesine-resistant cell lines by the acid guanidinium thiocyanate-phenol-chloroform extraction method (Chomczynski & Sacchi, 1987). Twenty μg of total RNA was electrophoresed on a 1% agarose-6% formaldehyde gel at 25 V for 12 h. The RNA was transferred to a positively charged nylon membrane (Hybond-N+, Amersham Japan), which was then hybridised overnight with a $^{32}$P-labelled DNA probe at 42°C. The probe was labelled with [α-$^{32}$P]-dCTP by means of a multiprime labelling system kit (Amersham Japan). After hybridisation, the membrane was washed three times with 2 × SSC (1 × SSC consists of 0.15 M NaCl and 0.015 M sodium citrate) and 0.1% SDS for 10 min. After 3 × washes with 0.1 × SSC and 0.1% SDS for 15 min at 65°C, followed by several washes with 0.1 × SSC, the membrane was autoradiographed on X-ray film (Amersham Hyperfilm-MP) at − 70°C for 3 days. The probe used, p*mdr*l (coding for human *mdr*l) (Roninson *et al.*, 1986), was kindly provided by I.B. Roninson (University of Ilinois).

*Preparation of whole cell lysates for determination of the total tubulin level*

Total tubulin was isolated by a modification of the method reported by Thrower *et al.* (1991) and Minotti *et al.* (1991). Exponentially growing cells were washed twice with PBS, collected and adjusted to $5 \times 10^5$ cells × $ml^{-1}$. One ml of each cell suspension was centrifuged at 200 *g* for 5 min, and resuspended in 0.3 ml of depolymerisation buffer (0.1 M MES, 1 mM $MgSO_4$, 10 mM $CaCl_2$, 5 mM GTP, pH 6.9). Cells were lysed by sonication on ice with a Branson Sonifier 450 (Branson ultrasonics, Danbury, CT) at 15 W output for two intervals of 15 s each. Lysate fractions were incubated in depolymerisation buffer for 1 h on ice to depolymerise the microtubules. Following depolymerisation, samples were centrifuged at 50,000 *g* for 15 min at 4°C using a TL-100 centrifuge (Beckman) with a fixed-angle TL-45 rotor. The supernatant was transferred to a new centrifuged tube. After addition of 30 μl protein G sephrose 4 Fast Flow (Pharmacia-LKB) and overnight mixing at 4°C, the tube was centrifuged at 250 *g* for 5 min. The supernatant was transferred to a new centrifuge tube, anti-α-tubulin antibody was added and the tube mixed for 1 h at 4°C. To each sample was then added 30 μl protein G sephrose followed by mixing for 1 h at 4°C and washing with 3 times depolymerisation buffer. To the pellet was added 50 μl SDS sample buffer (0.25 M Tris-HCl, 2% sodium dodecyl sulfate (SDS), 30% glycerol, 10% 2-mercaptoethanol, 0.01% bromophenol blue pH 6.8), and the mixture was then denatured at 90°C for 1 min and subjected to electrophoresis in a 10% SDS-polyacrylamide gel (PAG PLATE 10, Daiichi Pure Chemicals). The separated protein was stained using a silver stain (Sil-best Stain, Nacalai Tesque).

*Preparation of whole cell lysates for determination of the polymerised tubulin content*

Polymerised tubulin was isolated by a modification of the Thrower and Minotti method. Briefly, exponentially growing cells were washed twice with warm PBS, collected and adjusted to $5 \times 10^5$ cells × $ml^{-1}$. One ml of each cell suspension was centrifuged at 200 *g* for 5 min. The pellet was resuspended in 1 ml of stabilisation buffer (20 mM Tris-HCl, pH 6.8, 0.14 M NaCl, 0.5% Nonidet P-40, 1 mM $MgCl_2$, 2 mM EGTA, 4 μg $ml^{-1}$ taxol) and incubated at 37°C for 30 min. Each sample was centrifuged at 50,000 *g* for 15 min at 37°C. The supernatant was aspirated and the pellet was resuspended with 0.3 ml of depolymerisation buffer. Following this step the lysate was treated in a similar manner to the total tubulin isolates, with 1 h incubation on ice, centrifugation, immunoprecipitation, and electrophoresis.

*Western blot analysis for total and polymerised tubulin*

Total and polymerised tubulin contents were analysed on a 10% SDS-polyacrylamide gel. Following electrophoresis, the protein on the gel was electrophoretically transferred to nitrocellulose membranes (Towbin *et al.*, 1979). The membrane was incubated with blocking buffer (4% skimmed milk in PBS for 1 h and was then allowed to react with anti-α-tubulin antibody for 12 h at 4°C. After incubation, the membrane was washed four times with PBS containing 0.1% tween-20 and then incubated with biotinylated anti-mouse IgG antibody at room temperature for 1 h. The bands were detected by the ECL Western blotting detection method (Amersham), and analysed with a Ultroscan XL enhanced laser densitometer (Pharmacia LKB).

### Results

*General characteristics*

Data in Table I list the general characteristics of the parental H69 and the vindesine-resistant H69/VDS cell line. Cell diameter was measured using a Coulter chanelyser 256 (Nikkaki, Tokyo). The diameter of H69/VDS cells was slightly smaller than that of H69 cells, but the difference did not reach statistical significance (unpaired Student *t*-test, $P = 0.09$). The cell number doubling time of H69/VDS (62.3 h) was significantly longer than that of H69 (51.3 h) ($P < 0.05$). The protein contents of H69/VDS (292 μg $1 \times 10^{-6}$ cells) and H69 (270 μgl $1 \times 10^{-6}$ cells) were similar.

**Table I** Characteristics of H69 and H69/VDS

| | *H69* | *H69/VDS* |
|---|---|---|
| Diameter (μM)[a] | 8.10 ± 0.22[b] | 7.44 ± 0.74[c] |
| Doubling time (h) | 51.3 ± 5.8[d] | 62.3 ± 3.5[e] |
| Protein content (μg × $10^{-6}$ cells) | 270 | 292 |

[a]Diameters were measured using Coulter channeliser 256. [b]Each value is the mean ± s.d. of five independent experiments. [c]Each value is the mean ± s.d. independent experiments. There was no significant difference between the value of cell size for H69 and H69/VDS by unpaired Student *t*-test ([b] *vs* [c], P = 0.09). Significance in the difference of doubling time between H69 and H69/VDS by unpaired Student *t*-test ([d] *vs* [e], *P* = 0.048).

### *Growth-inhibitory effects of anticancer drugs in H69/VDS cells*

The growth-inhibitory effects of various drugs in H69 and H69/VDS cells were measured by the MTT assay and the $IC_{50}$'s are shown in Table II. The relative resistance of H69/VDS was highest against vindesine (× 11.6) and cross-resistance to vincristine (× 6.9), colchicine (× 3.4) and taxol (× 10.1) was evident ($P<0.01$, unpaired Student *t*-test). H69/VDS did not show high cross-resistance to other drugs such as doxorubicin (× 1.53), etoposide (× 0.87) or cisplatin (× 0.61). There were no significant differences in the $IC_{50}$'s value for these drugs between H69 and H69/VDS by unpaired Student *t*-test. The resistance pattern of H69/VDS cells was therefore different from that of the typical multidrug resistance phenotype. It was interesting to note that H69/VDS cells had cross-resistance not only to vincristine, colchicine but also to taxol, which has a different mechanism of action on tubulin compared with vinca alkaloids (Schiff *et al.*, 1979; Schiff & Horwitz, 1980).

### *Accumulation study*

To determine whether decreased drug accumulation was a cause of resistance, we examined the intracellular accumulation of [$^3$H]-VCR in H69 and H69/VDS cells. Table III shows the intracellular concentrations of [$^3$H]-VCR after the addition of 1, 5, and 10 nM [$^3$H]-VCR to the culture medium for 3 h. The amount of intracellular radioactivity of [$^3$H]-VCR increased in both H69 and H69/VDS cells with the increased concentration of [$^3$H]-VCR in the medium. There was no statistical difference in the intracellular [$^3$H]-VCR content of H69 and H69/VDS cells at any of the concentrations tested. Figure 1 shows that the time course of accumulation of [$^3$H]-VCR in H69 and H69/VDS cells was not significantly different. Similarly, there was no significant difference in the time course of efflux in H69 and H69/VDS cells (Figure 2). Etoposide-resistant H69/VP cells were used as positive controls as for typical *mdr*1 expressing cells (Minato *et al.*, 1990). In H69/VP cells the intracellular [$^3$H]-VCR retention was lower than in other cells (Figure 2). Table IV shows the accumulation of doxorubicin after 3 h exposure in H69, H69/VDS and H69/VP cells. There was no significant difference between the values for H69 and H69/VDS at each concentration. There was however a statistical difference in the concentration of doxorubicin between H69 and H69/VP cells. An enhancing effect of verapamil on the accumulation of doxorubicin in H69/VP was obtained. In H69 and H69/VDS however, no significant effect of verapamil on the accumulation of doxorubicin was observed. This result suggests that alteration of intracellular accumulation of [$^3$H]-VCR and doxorubicin was not responsible for the sensitivities to vincristine and doxorubicin in H69/VDS cells, respectively. H69/VDS did not show a typical multidrug resistant phenotype as determined by decreased cellular drug retention.

**Table II** Sensitivities to various agents in the MTT assay

| *Drug* | $IC_{50}$[a] (μM) *H69* | *H69/VDS* | *Relative resistance*[c] |
|---|---|---|---|
| VDS | 0.00167 ± 0.00009[b] | 0.0193 ± 0.0009 | 11.6[d] |
| Taxol | 0.0062 ± 0.0023 | 0.063 ± 0.020 | 10.1[d] |
| VCR | 0.0020 ± 0.0001 | 0.0137 ± 0.0012 | 6.85[d] |
| Colchicine | 0.00153 ± 0.00025 | 0.00514 ± 0.00015 | 3.36[d] |
| DOX | 0.150 ± 0.020 | 0.230 ± 0.060 | 1.53[e] |
| Etoposide | 5.627 ± 0.404 | 4.63 ± 0.67 | 0.87[e] |
| CDDP | 2.74 ± 1.31 | 1.66 ± 0.81 | 0.61[e] |

[a]Drug concentration that inhibits cell growth by 50%. [b]Each value is the mean ± s.d. of three or five independent experiments. [c]Relative resistance value equals the $IC_{50}$ value of resistant cell line divided by the $IC_{50}$ value of parental cell line. [d]$P<0.01$ (unpaired Student's *t*-test). [e]Not significant (unpaired Student's *t*-test).

**Table III** Accumulation of [$^3$H]-VCR for 3 h in H69 and H69/VDS cells

| *VCR (nM)* | *VER (μM)* | *Intracellular [$^3$H]-VCR (pmol × $10^{-6}$ cells)* *H69* | *H69/VDS* |
|---|---|---|---|
| 1 | 0 | 22.47±11.42[a,b] | 26.35±14.9[c] |
| 5 | 0 | 80.38±28.37[d] | 103.53±15.52[e] |
| 10 | 0 | 175.45±42.67[f] | 160.72±40.65[g] |
| 10 | 20 | 250.85±39.8[h] | 317.43±83.27[i] |

[a]Each value is the mean±s.d. of three independent experiments. There was no significant difference between the value for H69 and H69/VDS at each concentrations by unpaired Student's *t*-test. [b] *vs* [c], *P* = 0.817. [d] *vs* [e], *P* = 0.113. [f] *vs* [g], *P* = 0.55. [h] *vs* [i], *P* = 0.108. Significance in the difference of the concentration of [$^3$H]VCR in H69 and H69/VDS between VCR only and VCR + VER by unpaired Student *t*-test. [f] *vs* [h], *P* = 0.01. [g] *vs* [i], *P* = 0.002.

**Figure 1** Time course of cellular uptake and retention of [$^3$H]-VCR. H69 (open circle) or H69/VDS (closed square) cells were treated with 10 μM of drug.

**Figure 2** Time course of efflux of [$^3$H]-VCR in H69 (open circle), H69/VDS (open square), H69/VDS with 20 μM of verapamil (closed square) and H69/VP (triangle). After 3 h exposure with 10 μM of [$^3$H]-VCR with or without verapamil, cells were washed with drug free medium and [$^3$H]-VCR retained was examined at 30, 60 and 120 min.

**Table IV** Accumulation of DOX for 3 h in H69, H69/VDS and H69/VP cells

| | | *Intracellular DOX (μmol × $10^{-6}$ cells)* | | |
|---|---|---|---|---|
| *DOX (μM)* | *VER (μM)* | *H69* | *H69/VDS* | *H69/VP* |
| 10 | 0 | 1.22±0.18[a,b] | 1.50±0.28[c] | 0.87±0.15[d] |
| 100 | 0 | 3.36±0.35[e] | 3.21±0.61[f] | 1.74±0.10[g] |
| 100 | 20 | 3.31±0.29[h] | 3.26±0.36[i] | 2.35±0.22[j] |

[a]Each value is the mean±s.d. of three independent experiments. There was no significant difference between the value for H69 and H69/VDS at each concentration by unpaired Student's *t*-test. Significance in the difference of the concentration of DOX between H69 and H69/VP by unpaired Student *t*-test. [b] *vs* [d], $P<0.01$. [e] *vs* [g], $P<0.01$. [h] *vs* [j], $P<0.01$. [g] *vs* [j], $P<0.01$.

*Northern blot analysis*

To confirm that H69/VDS did not have P-glycoprotein mediated multidrug resistant phenotype, we examined the *mdr*1 mRNA expression in H69/VDS by Northern blot analysis. Doxorubicin resistant K562/ADM cells (Tsuruo *et al.*, 1986) and etoposide resistant H69/VP cells were used as positive controls for typical *mdr*1 expressing cells. The human *mdr*1 mRNA was not detected in H69/VDS cell line thus suggesting that the resistance of H69/VDS was not mediated by the *mdr*1 gene (Figure 3).

*Reversal of vindesine resistance by verapamil*

We examined the effect of $Ca^{2+}$-channel blocker, verapamil on vindesine cytotoxicity in H69 and H69/VDS cells. We also evaluated the toxicity of verapamil alone on H69 and H69/VDS cells using a dose range of 0.1 μM to 1,000 μM. We found that 20 μM of verapamil was not toxic to either cell line (Figure 4). The viability of both cell lines was more than 90% after 96 h exposure to 20 μM of verapamil in three independent experiments (Figure 4). $IC_{50}$'s for vindesine alone were 1.9 and 24.0 nM for H69 and H69/VDS, respectively (Figure 5). In the presence of 20 μM verapamil, the $IC_{50}$'s for vindesine in H69 and H69/VDS decreased to 0.5 and 2.0 nM415, respectively. Twenty μM of verapamil enhanced by 12-fold the growth-inhibitory effect of vindesine in H69/VDS cells. In H69 parental cells the corresponding sensitisation was only 3.8-fold, and therefore selective sensitisation by verapamil of the resistant cells was seen.

In order to determine whether verapamil influenced the intracellular accumulation of vincristine, H69 and H69/VDS cells were incubated with 10 nM of [$^3$H]-VCR in the presence or absence of 20 μM of verapamil for 3 h. Data in Table III show that, in the presence of verapamil, intracellular [$^3$H]-VCR content was increased from 175.4 to 250.9 pmol per $10^6$ cells, and from 160.7 to 317.4 pmol per $10^6$ cells in H69 and H69/VDS respectively. Verapamil increased the intracellular accumulation of [$^3$H]-VCR in both cell lines. However, in the presence of verapamil, the intracellular accumulation of [$^3$H]-VCR in H69/VDS cells was not significantly different from that in H69 cells. Verapamil did not increase the intracellular accumulation of doxorubicin in either H69 or H69/VDS cells, although it did so in H69/VP cells (Table IV). These results suggest that the reversal of vindesine-resistance in H69/VDS by verapamil could not be explained by an increase in the intracellular accumulation of vincristine. Considering that H69/VDS cells neither expressed *mdr*1 mRNA nor had reduced intracellular retention of vincristine reversal of vindesine-resistance by verapamil must be based on a mechanism other than decreased drug efflux induced by verapamil.

*Tubulin content*

We examined other mechanisms which might possibly be responsible for resistance to tubulin-acting agents in H69/VDS. As H69/VDS showed cross-resistance to vindesine, vincristine, colchicine, and taxol, drugs which are known to intereact with microtubules, we considered that changes in tubulin might be related to the resistance of H69/VDS cells. To determine whether verapamil affects the total and

**Figure 3** Northern blot analysis of *mdr*l in H69 and H69/VDS. The doxorubicin resistant cell line (K562/ADM) and etoposide-resistant cell line (H69/VP) were used as the positive control. **a**, The human *mdr*l mRNA (4.3 kb). **b**, Ethidium bromide-stained RNA.

**Figure 4** Cytotoxicity of verapamil in H69 and H69/VDS cells. H69 (open circle) and H69/VDS (closed square) cells were exposed with various concentration of verapamil for 96 h, the growth-inhibitory effect of verapamil was measured by the MTT assay from three independent experiments.

**Figure 5** The enhancement on the cytotoxicity of vindesine in presence (closed symbol) or absence (open symbol) of 20 μM of verapamil was evaluated in H69 (square) and H69/VDS (circle).

polymerised tubulins in H69/VDS and H69 cells, we measured by immunoprecipitation the total and polymerised tubulin contents after incubating cells in the presence or absence of 20 μM verapamil (Figure 6). The polymerised tubulin ratio is defined as the ratio of polymerised tubulin content to total tubulin content. The polymerised tubulin ratio in H69/VDS in the absence of verapamil was higher than that of H69. In H69, 20 μM of verapamil slightly increased the amount of total and polymerised tubulins, but the polymerised tubulin ratio remained unchanged. On the other hand, in H69/VDS, 20 μM of verapamil increased the amount of the total tubulin, but decreased the amount of polymerised tubulin resulting in a decrease in the polymerised tubulin ratio. This effect of verapamil on the total and polymerised tubulins in H69/VDS is consistent with the reversal of resistance by verapamil in H69/VDS. The decreased polymerised tubulin ratio caused by the addition of verapamil might explain the reversal of vindesine and vincristine resistance in H69/VDS.

*Dose and time dependent effects of verapamil on the amount of the total tubulin*

We observed two bands of 68 and 54 kDa by immunoprecipitation assay. The 68 kDa band was not detected by Western blot analysis and so this band was considered to be the tubulin-related protein. The 54 kDa band was identified as α-tubulin by reblotting. Total α-tubulin content of H69/VDS cells was significantly lower than that of the H69 parental cells (Figure 6). The time and dose dependent effects of verapamil on tubulin content were evaluated. The total tubulin content was increased in H69/VDS, as the concentration verapamil was increased from 5 to 10 20 μM (Figure 7a). The time-dependent change in the total tubulin content was evaluated in cells co-incubated with 20 μM of verapamil (Figure 7b). Increase in tubulin content caused by verapamil exposure was observed after 2 days. These results suggest that verapamil increased the tubulin content of H69/VDS cells in a both time- and dose-dependent manners.

**Figure 6** Influence of verapamil on the total and polymerised tubulin. H69 and H69/VDS cells were incubated with presence or absence of 20 μM of verapamil for 4 days, and total and polymerised tubulin contents of both cell lines were measured. Lane 1 indicated total tubulin content without verapamil in H69. Lane 2 indicated polymerised tubulin content without verapamil in H69. Lane 3 indicated total tubulin content with verapamil in H69. Lane 4 indicated polymerised tubulin content with verapamil in H69. Lane 5 indicated total tubulin content without verapamil in H69/VDS. Lane 6 indicated polymerised tubulin content without verapamil in H69/VDS. Lane 7 indicated total tubulin content with verapamil in H69/VDS. Lane 8 indicated polymerised tubulin content with verapamil in H69/VDS.

*Dose dependent effects of verapamil on polymerised tubulin ratio examined by Western blotting*

The dose dependent effects of verapamil on polymerised tubulin ratio were evaluated by Western blotting (Figure 8). The total tubulin content after exposure to 0.1 to 20 μM of verapamil was higher than that without verapamil in H69/VDS. Whereas the polymerised tubulin content was decreased. The polymerised tubulin ratio was therefore decreased by the co-incubation with verapamil.

### Discussion

Resistance to vinca alkaloids and taxol has been reported to involve the typical P-glycoprotein mediated multidrug resistance and increased drug efflux (Roy & Horwitz, 1985; Beck, 1987). In the vindesine resistant small-cell lung cancer cell line, H69/VDS which we established, cross-resistance to tubulin interacting agents such as vindesine, vincristine, colchicine and taxol was apparent (Table II). However, H69/VDS did not show cross-resistance to doxorubicin, etoposide and cisplatin (Table II). The resistant pattern of H69/VDS seemed to be different from that of the typical multidrug resistance phenotype. The expression of *mdr*1 mRNA was negative by Northern blot analysis in H69/VDS. The drug accumulation and efflux were equal in H69 and H69/VDS. Although verapamil could reverse the drug resistance in H69/VDS, the changes in accumulation of the drugs brought about by verapamil were the same in H69 and H69/VDS (Table III). The concentration (20 μM) of verapamil used in these experiments was not toxic to either of the cell lines. The viabilities of both cell lines were more than 90% after 96 h exposure with 20 μM of verapamil. Based on these data, it was strongly suggested that *mdr*1 gene expression is not

**Figure 8** Total and polymerised α-tubulin contents in H69/VDS by Western blotting with anti-α-tubulin antibody. H69/VDS cells were incubated by 0, 0.1, 0.5, 1 and 20 μM of verapamil for 4 days. AU stands for arbitrary units.

**Figure 7** Total α-tubulin contents in H69 and H69/VDS by immunoprecipitation by anti-α-tubulin antibody. The band of 68 kDa was the tubulin related protein and the band of 54 kDa (arrow) indicated α-tubulin content. **a**, H69/VDS cells incubated with 0, 5, 10, and 20 μM of verapamil for 4 days. **b**, H69/VDS cells incubated with 20 μM of verapamil for 0, 2, and 4 days.

involved for the resistance of H69/VDS. The site of action of vinca alkaloids is generally considered to be on the microtubules. We therefore considered that some changes in the tubulin might be related to the resistance of H69/VDS. By immunoprecipitation with anti-α-tubulin antibody for the comparison of the tubulin content of H69 and H69/VDS cells two bands of 68 and 54 kDa were observed. The 54 kDa band was identified as α-tubulin. On the other hand the 68 kDa band was not detected by Western blotting. At first we were interested in 68 kDa immunoprecipitated protein. There is a possibility that the 68 kDa immunoprecipitated protein is the 68 kDa microtubule associated protein (Lim *et al.*, 1984) or a heat-shock protein 70 (Lee *et al.*, 1992). However, the change in the amount of the 68 kDa immunoprecipitated protein was not reproducible in several experiments. Because of these findings, we did not consider that the 68 kDa protein was responsible for the resistance to vindesine in H69/VDS cells.

Verapamil increased the total cellular tubulin content (Figure 8). We investigated the synthesis of the total α-tubulin content using a [$^{35}$S]-methionine label to determine whether the effect of verapamil in increasing in the total tubulin content was due to an increase in synthesis. We did not, however, observe any increase in the synthesis of the total tubulin by the verapamil in H69/VDS (data not shown). We are now investigating the possibility that verapamil might influence the tubulin turnover.

As shown in the present study, the total and polymerised tubulin contents of H69/VDS were lower than those of parental H69 cells. The polymerised tubulin ratio in H69/VDS was higher than that of H69 and was decreased by the addition of verapamil in a dose-dependent manner (Figure 8). This result was consistent with the reversal of vindesine resistance in H69/VDS by verapamil. Therefore we believe that the increase in total tubulin and/or the decrease in the polymerised tubulin ratio plays some role in the mechanism of H69/VDS resistance. It has previously been reported that a decrease in the total cellular tubulin pool occurs parallel with an increase in microtubule depolymerisation (Jordan, 1991).

Taxol promotes the polymerisation of microtubules and stabilises tubulin polymers by preventing their depolymerisation. It seems that the manner of action of taxol on tubulin polymerisation is the reverse of that of vinca alkaloids. We therefore expected H69/VDS cells to show a hypersensitivity to taxol. However, H69/VDS cells showed cross-resistance to taxol. It has been reported that taxol does not competitively inhibit the binding of colchicine to tubulin (Schiff & Horwitz, 1981). This suggests that the binding site of taxol may be different from that of colchicine. The cytotoxic action and cytotoxic profile of taxol may not therefore be completely adverse to those of vinca alkaloids. We thought it to be not unreasonable therefore that H69/VDS did not show hypersensitivity to taxol. However, it remains unclear why H69/VDS did, in fact show cross-resistance to taxol. Further investigations are necessary to clarify this question.

Verapamil is known for its capacity to circumvent the typical multidrug resistance phenotype by reducing the increased drug efflux in the resistant cells (Yusa & Tsuruo, 1989). On the other hand, verapamil has also been reported to potentiate drug effects in the absence of P-glycoprotein (Nygren & Larsson, 1990). The effect of verapamil on tubulin, presented here, might indicate a new mode of action in circumvention of vinca alkaloid resistance other than by interaction with P-glycoprotein.

### References

BECK, W.T. (1987). The cell biology of multiple drug resistance. *Biochem. Pharmac.*, **36**, 2879–2887.

BUNN, P.A. Jr, CULLEN, M., FUKUOKA, M., GREEN, M.R., HANSEN, H.H., HARPER, P., JOHNSON, D., KLASTERSKY, J., CHEVALIER, T.L., SAGMAN, U. & SPLINTER, T. (1989). Chemotherapy in small cell lung cancer: a consensus report. *Lung Cancer*, **5**, 127–134.

CHOMCZYNSKI, P. & SACCHI, N. (1987). Single-step method of RNA isolation by acid guanidinium thiocyanate-phenol-chloroform extraction. *Anal. Biochem.*, **162**, 156–159.

HORICHI, N., TAPIERO, H., SUGIMOTO, Y., BUNGO, M., NISHIYAMA, M., FOURCADE, A., LAMPIDIS, T.J., KASAHARA, K., SASAKI, T., TAKAHASHI, T. & SAIJO, N. (1990). 3′-deamino-3′-morpholino-13-deoxo-10-hydroxycarminomycin conquers multidrug resistance by rapid influx following higher frequency of formation of DNA single- and double-strand breaks. *Cancer Res.*, **50**, 4698–4701.

JORDAN, M.A., THROWER, D. & WILSON, L. (1991). Mechanism of inhibition of cell proliferation by vinca alkaloids. *Cancer Res.*, **51**, 2212–2222.

LAI, S.L., GOLDSTEIN, L.J., GOTTESMAN, M.M., PASTAN, I., TSAI, C.M., HOHNSON, B.E., MULSHINE, J.L., IHDE, D.C., KAYSER, K. & GAZDER, A.F. (1989). *Mdr*1 gene expression in lung cancer. *J. Natl Cancer Inst.*, **81**, 1144–1150.

LEE, W.-C., LIN, K.-Y., CHEN, K.-D. & LAI, Y.-K. (1992). Induction of HSP 70 is associated with vincristine resistance in heat-shocked 9L rat brain tumor cells. *Br. J. Cancer*, **66**, 653–659.

LIM, L., HALL, C., LEUNG, T. & WHATLEY, S. (1984). The relationship of the rat brain 68 kDa microtubule-associated protein with synaptosomal plasma membranes and with the *Drosophilia* 70 kDa heat-shock protein. *Biochem. J.*, **244**, 677–680.

MINATO, K., KANZAWA, F., NISHIO, K., NAKAGAWA, K., FUJIWARA, Y. & SAIJO, N. (1990). Characterization of etoposide-resistant human small-cell lung cancer cell line. *Cancer Chemother. Pharmacol.*, **26**, 313–317.

MINOTTI, A.M., BARLOW, S.B. & CABRAL, F. (1991). Resistance to antimitotic drugs in Chinese hamster ovary cells correlates with changes in the level of polymerized tubulin. *J. Biol. Chem.*, **266**, 3987–3994.

MOSMANN, T. (1983). Rapid calorimetric assay for cellular growth and survival: application of proliferation and cytotoxicity assay. *J. Immunol. Methods*, **65**, 55–63.

NYGREN, P. & LARSSON, R. (1990). Verapamil and cyclosporin A sensitize human kidney tumor cells to vincristine in absence of membrane P-glycoprotein and without apparent changes in the cytoplasmic free $Ca^{2+}$ concentration. *Biochem. Pharmacol.*, **10**, 231–237.

RONINSON, I.B., CHOI, K., GROS, P., HOUSMAN, D.E., FOJO, A., SHEN, D.W., GOTTESMAN, M.M. & PASTAN, I. (1986). Isolation of human *mdr* DNA sequences amplified in multi-resistant KB carcinoma cell lines. *Proc. Natl Acad. Sci. USA*, **83**, 4538–4542.

ROY, S.N. & HORWITZ, S.B. (1985). A phosphoglycoprotein associated with Taxol resistance in J774.2 cells. *Cancer Res.*, **45**, 3856–3863.

SCHIFF, P.B., FANT, J. & HORWITZ, S.B. (1979). Promotion of microtubule assembly *in vitro* by taxol. *Nature*, **227**, 665–667.

SCHIFF, P.B. & HORWITZ, S.B. (1980). Taxol stabilizes in mouse fibroblasts cells. *Proc. Natl Acad. Sci. USA*, **77**, 1561–1565.

SCHIFF, P.B. & HORWITZ, S.B. (1981). Taxol assembles Tubulin in the absence of exogenous guanosine 5′-triphosphate or microtubule-associated proteins. *Biochemistry*, **20**, 3247–3252.

THROWER, D., JORDAN, M.A. & WILSON, L. (1991). Quantitation of cellular tubulin in microtubules and tubulin pools by competitive ELISA. *J. Immunol. Methods*, **136**, 45–51.

TOWBIN, H., STAEHELIN, T. & GORDON, J. (1979). Electrophoretic transfer of proteins from polyacrylamide gels to nitrocellulose sheets. *Proc. Natl Acad. Sci. USA*, **76**, 4350–4354.

TSURUO, T., IIDA, SAITO, H., KAWABATA, H., OH-HARA, T., HAMADA, H. & UTAKOJI, T. (1986). Characteristics of resistance to adriamycin in human myelogenous leukemia K562 resistant to adriamycin and in isolated clones. *Jpn. J. Cancer Res.*, **77**, 682–692.

YUSA, K. & TSURUO, T. (1989). Reversal mechanism of multidrug resistance by verapamil: direct binding of verapamil to P-glycoprotein on specific sites and transporst of verapamil outward across the plasma membrane of K562/ADM cells. *Cancer Res.*, **49**, 5002–5006.

VERSANTVOORT, C.H.M., BROXTERMAN, H.J., PINEDO, H.M., DE VRIES, E.G.E., FELLER, N., KUIPER, C.M. & LANKELMA, J. (1992). Energy-dependent processes involved in reduced drug accumulation in multidrug resistant human lung cancer cell lines without P-glycoprotein expression. *Cancer Res.*, **52**, 17–23.